# The Effect of Topical Anaesthesia on the Cortisol Responses of Calves Undergoing Dehorning

**DOI:** 10.3390/ani10020312

**Published:** 2020-02-17

**Authors:** Crystal Espinoza, Sabrina Lomax, Peter Windsor

**Affiliations:** 1 School of Life and Environmental Sciences, Faculty of Science, The University of Sydney, Sydney 2006, Australia; crystal.espinoza@sydney.edu.au; 2 Sydney School of Veterinary Science, Faculty of Science, The University of Sydney, Sydney 2006, Australia; peter.windsor@sydney.edu.au

**Keywords:** dehorning, calves, pain, topical anaesthesia, welfare

## Abstract

**Simple Summary:**

Dehorning in cattle involves the manual removal of horns which causes acute pain. Although the long-term solution to removing horns is to breed polled cattle, limitations include the complex inheritance for polled Brahman cattle, and negative interactions with productivity in dairy cattle. Best practice pain relief in the form of a local nerve block, a sedative and a non-steroidal anti-inflammatory drug prior to the procedure usually requires a veterinarian and may be impractical for some cattle production systems. Improved livestock welfare requires a practical and cost-effective pain relief strategy for dehorning that can be readily adopted commercially. We evaluated a modified topical local anaesthetic wound formulation that can be applied by farmers immediately following dehorning. While previous studies have demonstrated a reduction in wound sensitivity, there was no effect on cortisol concentrations. It is likely that the cortisol response reflects a combination of factors including the stress of handling, the initial pain of the dehorning procedure and haemorrhage.

**Abstract:**

Dehorning causes pain and distress to cattle, and there is a need to provide effective and practical analgesia to improve animal welfare. We conducted an experiment to determine the effect of a modified post-operative topical wound management formulation containing two local anaesthetics (TA) on the plasma cortisol concentration (PCC) of scoop-dehorned calves. Two months old Holstein-Friesian heifer calves (*n* = 30) were randomly allocated to sham dehorning control (CON), scoop dehorning (D), or scoop dehorning with immediate post-operative application of the TA (DTA). Blood samples were obtained via jugular venepuncture prior to sham or actual dehorning, and 40 min, 1.5, 4 and 24 h later. PCC changed significantly over time (*p* < 0.01). There was a trend for lower PCC in DTA calves compared to D calves (*p* = 0.09), with the PCC area under the curve lowest in CON calves as compared to D and DTA calves (*p* = 0.02). Cortisol concentrations were similar between D and DTA at all time points. The TA did not reduce cortisol concentrations up to 24 h following treatment and the cortisol response likely reflects the pain induced by the procedure, the effect of handling and restraint, and haemorrhaging which limited adherence of the TA actives. A multimodal analgesic approach, as assessed through multiple pain indicators, should be the focus of future work.

## 1. Introduction

Dehorning is a routine husbandry procedure in many commercial dairy and beef production enterprises which involves the removal of the horn and horn-producing tissue in genetically horned breeds of cattle. Cattle are dehorned to enable safe handling, to reduce injury to other cattle, and to reduce subsequent carcass bruising that reduces meat quality [1,2]. Cattle may also be dehorned to minimise the amount of trough space required at feeding [3]. Although dehorning remains a short-term solution for managing these issues, it is a procedure that will likely continue unless all farmed cattle become polled and dehorning is no longer required [4]. Recently, genetic research and commercial testing have successfully identified homozygous polled alleles to facilitate the selective breeding of polled cattle [5,6].

Extensive studies indicate that all three methods of dehorning including cautery, the use of caustic substances, and amputation, cause acute and chronic pain [2,7]. Amputation dehorning is the most common method of dehorning following attachment of the horn-bud to the skull, typically occurring after 2 months of age [8]. This method has been shown to cause a significant increase in cortisol secretion which persists for 7 to 9 h following dehorning, in the absence of anaesthesia or analgesia [9,10]. Similarly, changes in calf behaviour, including increased frequency of head shaking, ear flicking, tail flicking, and reduced rumination, are also indicative of a painful experience that persists for up to 6 h following dehorning when no anaesthesia or analgesia was provided [11]. Growing public concern for the welfare of production animals, has led to comprehensive research into different pain relief regimes for dehorning.

The pre-procedural sub-cutaneous administration of a local anaesthetic to block neurotransmission of the cornual nerve, combined with the intravenous administration of a non-steroidal anti-inflammatory drug, often accompanied with an intravenous or intramuscular injection of a sedative for improved restraint, is considered to provide the most effective means for addressing the pain of scoop dehorning [7,12,13]. This is performed by veterinarians, requiring skills enabling injection into the jugular and into the vicinity of cornual nerve, plus authorisation to purchase and administer regulated drugs. To allow time for the local anaesthetic to achieve efficacy and the NSAID to be adequately distributed, they should be administered approximately 20 min before dehorning occurs [12,13]. As this requires double-handling and additional time for completion of the procedure, dehorning has traditionally been performed without pain relief in many countries [14]. Producers and practitioners cite time, cost, and lack of information or skill, as some of the reasons for the lack of pain relief [14,15].

Laws concerning the use of pain relief for dehorning pain vary between countries. In Australia, there is no legislation mandating the use of pain relief when dehorning. All Australian states must follow the non-mandatory recommendations set by “The Model Code of Practice for the Welfare of Animals: Cattle” which recommends calves over 6 months of ages be treated with pain relief [16]. Recent legislative changes particularly in other developed countries have mandated the use pre-operative pain relief for dehorning [17]. New Zealand has legislated that from October 2019, local anaesthetic must be used prior to disbudding or dehorning cattle [18].

Farmer-applied topical local anaesthesia (TA) may address the cost, time and practical limitations of currently available methods of providing pain relief for dehorning [19]. TA enables a single application to be administered immediately after dehorning, removing the need to double-handle animals. As it can be safely applied by the farmer, the cost of requiring a veterinarian to dehorn the cattle is eliminated. Topical local anaesthetics are applied to the skin surface (less absorption) or directly onto the mucosal tissue or open wound to induce rapid and lasting local anaesthesia [20,21]. We have previously reported on a topical anaesthetic wound dressing formulation reducing short-term pain sensitivity and increasing mechanical nociceptive thresholds following scoop dehorning in calves [22,23]. This multipurpose formulation contains lignocaine and bupivacaine for local anaesthesia, cetrimide for antisepsis, and aluminium chlorohydrate for astringency of the wound (modified from Tri-Solfen^®^; Bayer Animal Health, Pymble, NSW, Australia). These ingredients are carried in a viscous gel base with the intent to improve adhesion to the dehorning wound and surrounding tissue. The assessment of pain and the efficacy of analgesics can be improved by using a combination of behavioural and endocrine measures [24]. Our previous research has used sensory testing and algometry as pain indicators for the assessment of TA efficacy [22,23]. The activity of the hypothalamic-pituitary-adrenocortical (HPA) system has been extensively measured to gauge the distress of dehorning and the effectiveness of analgesics [9,10,25,26]. Plasma cortisol concentrations provide an indication of HPA activity and are useful indices as, within certain limits, secretion occurs in a graded way in response to the presumed noxiousness of different experiences [27,28]. Amputation dehorning causes a typical pattern in cortisol secretion which peaks 30 min post dehorning, decreases to a plateau, then returns to pre-treatment levels by 6–7 h post dehorning [9,10,13,25]. The measurement of cortisol is the most commonly used physiological indicator of distress in cattle and is often used as an indirect indicator of pain-induced stress [7,27,29], with changes to its typical secretory pattern having facilitated the evaluation of different analgesic regimes [9,25,26]. Previous research has demonstrated the effect of analgesia and anaesthesia for reducing the cortisol response to dehorning [13,25,26,30,31].

The aim of this experiment was to assess the plasma cortisol response of Holstein-Friesian calves to scoop dehorning following treatment of the wounds with the topical anaesthetic wound management formulation described.

## 2. Materials and Methods

### 2.1. Animals and Housing

The experimental protocol was approved by the Animal Ethics Committee of the University of Sydney (Approval No. 5832) and was conducted in accordance with the guidelines of the ‘Australian code for the care and use of animals for scientific purposes’ [32]. Thirty Holstein-Friesian heifer calves (8 weeks ± 1 week) were sourced from the commercial replacement herd at ‘Corstophine Dairy’ at The University of Sydney Camden campus farms in New South Wales, Australia. Calves were raised under commercial dairy operational conditions prior to the experiment. As per standard farm practice, the calves were separated from their mothers by 2 d of age and were thereafter group-housed in 1 ha paddocks before and during the experimental period. Calves were group-fed a milk ration at 10% of their body weight twice a day at 07:30 h and 15:30 h via an artificial teat, and were provided ad-libitum access to water and kikuyu-based pasture, and small amounts of calf grower pellets. Concurrent to the cortisol samples collected for this study, animals underwent quantitative sensory testing (QST) as part of a previously published experiment [22]. QST, using von Frey monofilaments (instruments which depending on length and diameter, apply a specific calibrated force to a skin surface [22]), has been used in previous studies to assess different analgesic interventions in humans and animals [21,33,34]. Two weights of von Frey monofilaments (10 and 300 g) were used to mechanically stimulate 6 sites within and adjacent to the dehorned site. Behavioural responses were observed and categorised as either absent, minor, moderate or severe [22].

### 2.2. Experimental Design and Treatments

The experiment was blocked across 3 days, with 10 calves treated each day (Table 1). This ensured all calves were dehorned in the morning within 2.5 h and allowed sufficient time for all sampling to be completed. Within each block, the same protocol was repeated, and all treatments were represented.

On the morning of each day of experimentation, calves were quietly moved from their paddocks to a holding yard and then individually into a spin-roll calf cradle (Arrow Farmquip, Tamworth, NSW, Australia) and placed into right lateral recumbency for treatment and sampling. Calves were randomly assigned to 1 of 3 treatments: sham scoop dehorning (control group, CON; *n* = 10); scoop dehorning (D; *n* = 10); and scoop dehorning with an application of a topical anaesthetic gel (Bayer Animal Health, Gordon, NSW, Australia) (DTA; *n* = 10). Sham dehorning was performed by manually manipulating and placing scoop dehorners over each horn bud for 3 s without incising the tissue. Dehorning was performed by placing small sharpened scoop dehorners (33 cm Supa-Scoop Dehorner, Farmhand, Shoof International Pty Ltd., Melbourne, Australia) over the horn bud region and pulling apart the handles of the device to excise the horn bud and immediate surrounding skin. Sham dehorning or dehorning took place between 08:30 and 11:00 h. DTA calves received an immediate application (within seconds from dehorning) of topical anaesthetic gel (TA) to the wound and surrounding cut tissue. Approximately 4 mL of TA was applied to each dehorning wound (total of 8 mL per calf) with a sterile, soft silicone brush. The TA was carried in a viscous solution, and a depth of approximately 2 to 3 mm was applied over the wound and adjacent tissue. The brush was cleaned between calves in a disinfectant solution (Hibitane^®^, Coopers^®^ Animal Health, Intervet Pty Ltd., Bendigo East, VIC, Australia). Following treatment and data collection, calves were returned to their pens until subsequent data collection was required, where they were moved back to the holding yard and cradle.

### 2.3. Topical Anaesthetic Formulation

The TA contained two local anaesthetics, lignocaine hydrochloride (100.0 g/L) and bupivacaine hydrochloride (5.0 g/L), aluminium chlorohydrate (100.0 g/L) and cetrimide (5.0 g/L). This formulation was modified from a commercial product developed for mulesing wounds in lambs (Tri-Solfen^®^, Bayer Animal Health, NSW, Australia) [21] and now registered in Australia for a range of husbandry procedures in sheep and cattle, including disbudding and dehorning [19]. The lignocaine concentration was doubled from the original formulation to produce a more concentrated wound formulation that was considered more suitable for the smaller surface area of the dehorning wound. Aluminium chlorohydrate was included with an intention of promoting haemeostasis in response to the moderate to excessive volumes of blood loss often induced by scoop dehorning. The viscosity of the gel was also increased with an intention to improve adherence of the TA to the wound.

### 2.4. Blood Collection and Analysis

Calves were concurrently subjected to QST prior to treatment and at 1 and 40 min, 1.5, 4 and 24 h post-treatment as previously reported [22]. Blood samples for the current study were obtained before QST at each time point. Calves were restrained for an average of 3.5 min (range 2–6 min) for blood and data collection.

Upon placement into lateral recumbency, a 5 mL blood sample was drawn from the jugular vein using an 18 G needle into a 9 mL EDTA vacutainer. The tube was inverted several times and placed on ice until centrifugation 7–9 h later. Further blood samples were collected at 40 min and 1.5, 4 and 24 h post treatment. Blood samples were centrifuged at 3000 RPM for 15 min and the separated plasma layer pipetted off into a labelled and sterile 5 mL serum vial. Samples were stored at −80 °C pending analysis.

Plasma cortisol concentrations were determined in duplicate using the commercially available radio-immunoassay kit (Coat-a-Count^®^ Cortisol RIA; Siemens Pty Ltd., Los Angeles, CA, USA). The lowest detectable concentration was 6.9 nmol/L and any samples below this threshold were denoted as 0 nmol/L. The intra-assay and inter-assay coefficients of variation were 4.4% and 5.8% respectively.

### 2.5. Statistical Analysis

Data were analysed to determine the effect of treatment on plasma cortisol concentration. The raw data had near normal distribution and constant variance and did not require transformation. A residual maximum likelihood, linear mixed models (REML) analysis was conducted using the statistical software package, Genstat^®^ (18th edition, VSN International Ltd., Hemel Hempstead, UK). The fixed effects of the model were treatment (CON, D, DTA), time (pre-treatment, 40 min, 1.5 h, 4 h, 24 h post-treatment), and their interaction. The random effect of the model was calf ID.

The area under the curve with respect to the ground (AUC) [35] was examined to determine the magnitude and severity of the cortisol response to treatment over the 24 h period post-dehorning. AUC was calculated using the linear trapezoidal method [35] for the following time intervals: 0–24 h (AUC_0–24 h_), 0–40 min (AUC_0–40 min_), 40 min–1.5 h (AUC_40 min–1.5 h_), 1.5–4 h (AUC_1.5–4 h_), and 4–24 h (AUC_4–24 h_). Zero cortisol concentrations were modified to 3.4 nmol/L (midpoint between 0 and 6.9 nmol/L) to facilitate AUC calculations. Data were log_e_ transformed to attain normal distribution and constant variance. In Genstat^®^, a one-way analysis of variance (ANOVA) was used to determine treatment mean differences. For all statistical calculations, *p* ≤ 0.05 were considered statistically significant and pair-wise comparisons with differences greater than the corresponding least significant difference (LSD) were considered statistically significant. *p* > 0.05 ≤ 0.1 were considered as statistical tendencies.

## 3. Results

**Figure 1:**
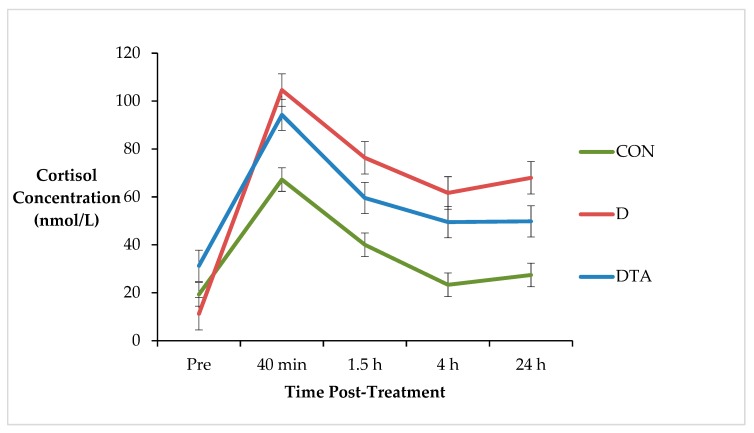
Cortisol concentration (nmol/L; ± s.e.m.) of calves subjected to sham dehorning (CON), dehorning (D) and dehorning with an application of topical anaesthetic (DTA) prior to treatment and at 30 min, 1.5 h, 4 and 24 h post-treatment.

Two DTA calves were removed from the raw cortisol analysis due to missing data (CON, *n* = 10; D, *n* = 10; DTA, *n* = 8). There was a statistical tendency for a treatment x time interaction for cortisol concentration (*p* = 0.086; s.e.d. = 11.75) (Figure 1). There was no difference between treatments pre-dehorning and the lowest cortisol response was observed in CON group (*p* = 0.086). DTA calves tended to have cortisol concentrations intermediate to CON and D calves.

**Figure 2:**
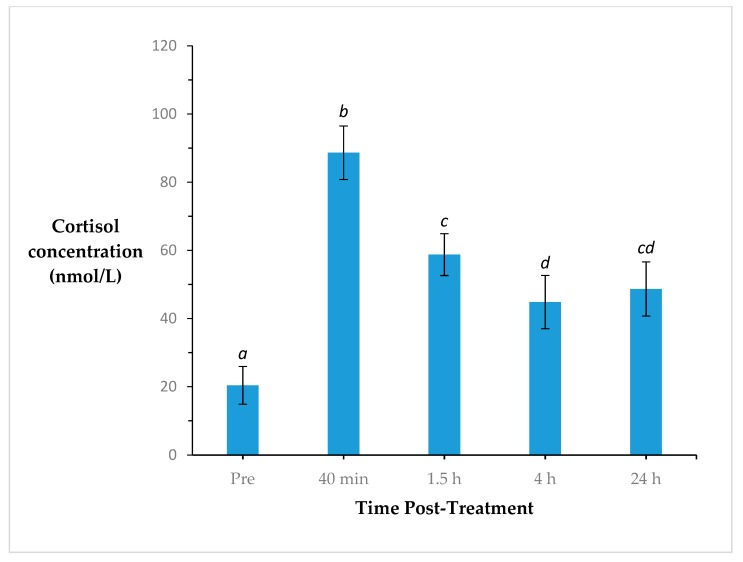
Cortisol concentration (nmol/L ± s.e.m.) of all calves prior to treatment, and at 40 min, 1.5 h, 4 and 24 h post-treatment. Time points with different superscripts are significantly different (*p* < 0.05).

Plasma cortisol concentration changed significantly over time (*p* < 0.001; s.e.d. = 6.946) (Figure 2). Cortisol concentrations were lowest prior to treatment than at any other time point. Cortisol peaked at 40 min post-treatment and decreased thereafter. At 24 h post-treatment, cortisol concentrations were intermediate to cortisol concentrations at 1.5 and 4 h post-treatment.

**Figure 3:**
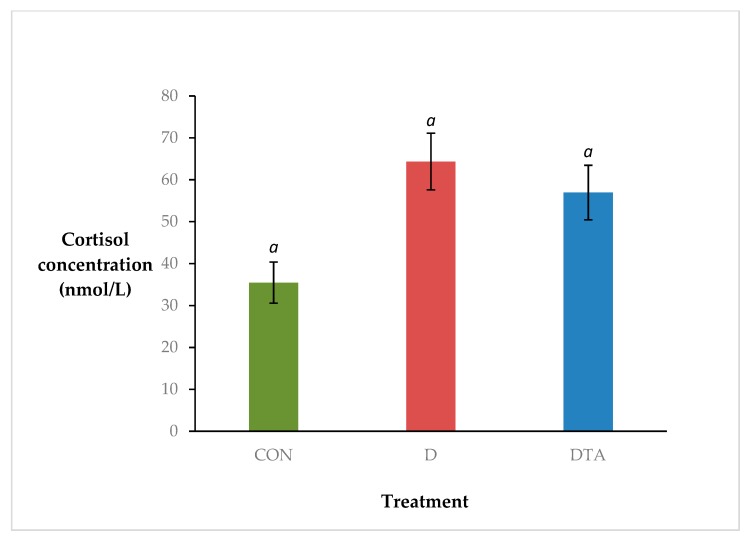
The effect of treatment (sham dehorning, CON; dehorning, D; dehorning with topical anaesthetic, DTA) on cortisol concentration (nmol/L; ± s.e.m.). There was no significant effect of treatment on cortisol concentration (*p* = 0.079).

There was a statistical tendency for a treatment effect on cortisol response (*p* = 0.079; s.e.d. = 12.88) (Figure 3). Overall, the lowest cortisol concentrations were secreted by CON calves, the highest from D calves, and DTA calves were intermediate to both (mean 35, 64 and 57 nmol/L, respectively).

One DTA calf was removed from the AUC analysis as it had a large residual (CON, *n* = 10; D, *n* = 10; DTA, *n* = 7). AUC_0–24 h_ was significantly lower in CON calves than D and DTA calves (*p* = 0.02) (Table 2). There was no significant difference in AUC_0–40 min_ between all treatment groups (*p* = 0.07). There was no significant difference in AUC between D and DTA calves at any time period (*p* > 0.05).

## 4. Discussion

The results of this experiment demonstrate the plasma cortisol response to surgical dehorning, with peak concentration occurring 40 min post procedural intervention. There was a clear effect of handling and procedural pain on cortisol response, with no difference in plasma cortisol concentrations (PCC) between the two cohorts of surgically dehorned calves.

We identified statistical trends for lower PCC and AUC in DTA compared to D calves. In addition, the PCC of dehorned calves exceeded that of sham dehorned calves. This suggests that timing of anaesthetic delivery may partly account for the observed cortisol response. Blockage of local nociception after the administration of a noxious stimuli is unlikely to greatly influence the HPA response, particularly as the elevated cortisol response presumably reflects both the stress of prolonged restraint plus haemorrhage from the wound site [36,37,38].

There was no significant difference in AUC_0–40 min_ between sham dehorned calves and dehorned calves regardless of treatment with TA, indicating the stress of handling and restraint on cortisol response [36]. The stress of sham dehorning has been well documented and causes a small yet significant rise in cortisol concentrations from base level [9,10,13,25,39,40]. This is due to the activation of the HPA system by a number of external stressors including restraint, handling and the presence of humans [36,41]. In the current study, there was no significant difference in peak cortisol concentration between sham dehorned and dehorned calves, which contrasts previous dehorning studies [9,10,13,25]. This difference may be attributed to the type and duration of restraint used in this study. In other experiments, calves were manually restrained for a relatively short period of time in a standing position (<15–45 s) [9,10,13,25,39,40], whereas in the present study, calves were restrained in a spin-roll calf cradle that placed them into right lateral recumbency for a longer period of time (mean = 3.5 min, range = 2–6 min). While other experiments tended to only restrain for a quick blood collection, the present study required additional restraint time for QST for a concurrent study [22], which involved mechanical stimulation of the wound with a von Frey filament. While blood-sampling occurred immediately upon restraint, multiple testing may have induced additional pain and stress, and prolonged the cortisol response up to 24 h where PCC did not return to baseline levels.

A similar study investigating the effect of the original TA formulation (Tri-Solfen^®^ Bayer HealthCare Animal Health Inc., Sydney, Australia) for castration of 3-mo Angus bull calves reported a similar effect of handling and procedural pain on cortisol response [42]. There was no difference in cortisol concentration at 0.5 or 1 h post-castration with or without post-operative TA application [42]. The effect of repeated handling on cortisol response is well documented [43,44,45]. The results of this study and the current study indicate the importance of habituation to handling prior to evaluating cortisol response. This is further evident when considering the results from two studies evaluating the effect of TA for surgical castration [46] and hot-iron tail-docking and surgical mulesing [47] in lambs. In both studies, TA significantly reduced the cortisol concentration 30 min post procedure as compared to untreated lambs [38]. The key difference is that lambs were intensively habituated to handling for 2 weeks prior to the commencement of the study [46,47]. This presents a major limitation to using cortisol as a measure of pain, particularly when working under commercial conditions, as per the current study where the opportunity to habituate calves was limited.

The effect of TA administration on cortisol secretion may also depend on the type of tissue damage induced by the surgical procedure. Scoop dehorning in younger calves typically creates bony wounds of small surface area, with haemorrhage, which may limit the quantity of TA absorption. Haemorrhage reduces the ability of the TA to adhere and remain on the wound and has been observed previously [23]. The lack of epithelial or mucosal tissue on small bony dehorning wounds means there is minimal surface area for effective TA absorption, with most nociceptors likely located in the wound edges. Though the more viscous formulation developed for this experiment improved wound adherence, it did not eliminate bleeding. While TA has been shown to significantly reduce peak cortisol concentration in surgically mulesed lambs, mulesing wounds are generally superficial and have a much larger surface area for absorption [47]. A similar reduction in peak cortisol concentration was observed in surgically castrated lambs treated with TA with efficacy likely attributable to the fast and effective anaesthesia of some of the scrotal nerves and mucosal tissue [46]. However, in neither study, did TA application reduce the elevated cortisol response to the levels of sham handled animals [46]. Further, the AUC of TA-treated mulesed lambs was similar or greater than non-treated lambs up to 24 h post-treatment with the authors recommending a combined analgesic strategy for an improved welfare outcome [47].

The use of multiple pain indicators is important for a multi-faceted evaluation of pain and analgesic efficacy. The concurrent study evaluated the QST response of calf dehorning wounds treated with TA as a measure of nociception [22]. We demonstrated a significant reduction in nociception at 40 min and 1.5 h post-dehorning in calves treated with TA as compared to untreated calves. However a correlated reduction in cortisol concentration in this time period was not indicated in the current study. As QST measures nociception through mechanical stimulation of the wound surface, it provides a direct measurement of anaesthesia [21,33,34]. Cortisol responses, when used independently of other measures, should be interpreted with caution as they are not a direct measurement of pain, but an indication of the overall noxiousness associated with an experience, including emotional and physical components [48]. Its limitations as an indicator of pain assessment include high individual variation, diurnal changes, and the wide variety of physical and emotional experiences both pleasant and unpleasant that activate the HPA system [24,27]. Further, cortisol plays an important role in maintaining blood volume and may rise even in the absence of pain, including during surgical procedures under general anaesthesia [37,38].

Calves in the current study were not provided any pre-operative anaesthesia or analgesia, and therefore were completely nociceptive during the dehorning procedure. This is significant given that the combination of restraint and activation of damaged nerves at and shortly after severance of the horn-bud is the likely major cause of the initial cortisol peak [13]. Thus the provision of pre-operative anaesthesia to eliminate procedural nociception should be evaluated in combination with TA.

## 5. Conclusions

The application of a modified topical local anaesthetic formulation immediately after scoop dehorning in young dairy calves did not reduce cortisol concentrations up to 24 h following treatment. The cortisol response in this study likely reflects a combination of factors, including the pain induced by the procedure, the effect of handling and restraint, and presence of haemorrhage that may have inhibited efficacious long-term adherence of the TA actives, diminishing the potential pain relief provided. Future work should focus on multi-modal anaesthesia and multiple objective measures of pain.

## Figures and Tables

**Table 1:** Number of animals in each block and treatment group (sham scoop dehorning, CON; scoop dehorning, D; and scoop dehorning with topical anaesthetic, DTA).

		Treatment Group			**Block Total (n)**
		CON (*n*)	D (*n*)	DTA (n)	
**Block**	1	4	3	3	10
	2	3	4	3	10
	3	3	3	4	10
**Treatment group total (n)**		10	10	10	

**Table 2:** Area under the curve (AUC) (nmol/L per ha ± s.e.m.) for the plasma cortisol of calves subjected to sham dehorning (CON), dehorning (D), or dehorning with an application of topical anaesthetic (DTA). AUC_0–24_ = area under curve from pre-treatment to 24 h post-treatment; AUC_0–40 min_ = area under the curve from pre-treatment to 40 min post-treatment; AUC_40 min–1.5 h_ = area under the curve from 40 min to 1.5 h post-treatment; AUC_1.5–4 h_ = area under the curve from 1.5 h to 4 h post-treatment; AUC_4–24 h_ = area under the curve from 4 h to 24 h post-treatment. Within a row, means without a common superscript differ significantly (*p* < 0.05).

AUC Time Period	CON		D		DTA		*p*-Value
	Mean	s.e.m.	Mean	s.e.m.	Mean	s.e.m.	
AUC_0–24 h_	514.7 ^a^	160.5	1307.9 ^b^	331.8	1317.5 ^b^	299	0.02
AUC_0–40 min_	23.7	6.2	37.6	3.9	42.5	10.7	0.07
AUC_40 min–1.5 h_	39.0 ^a^	7.8	70.7 ^b^	9.7	66.1 ^b^	10.9	0.02
AUC_1.5–4 h_	82.0 ^a^	20.2	181.5 ^b^	37.2	169.6 ^b^	35.8	0.01
AUC_4–24 h_	330.6 ^a^	143.8	988.9 ^b^	284.3	1030.1 ^b^	249.2	0.02

a,b mean without a common superscript differ significantly (*p* < 0.05).
